# Managing Cancer and Living Meaningfully (CALM) alleviates chemotherapy related cognitive impairment (CRCI) in breast cancer survivors: A pilot study based on resting‐state fMRI

**DOI:** 10.1002/cam4.6285

**Published:** 2023-07-06

**Authors:** Senbang Yao, Qinqin Zhu, Qianqian Zhang, Yinlian Cai, Shaochun Liu, Lulian Pang, Yanyan Jing, Xiangxiang Yin, Huaidong Cheng

**Affiliations:** ^1^ Department of Oncology The Second Affiliated Hospital of Anhui Medical University Hefei China; ^2^ Cancer and Cognition Laboratory Anhui Medical University Hefei China; ^3^ Department of Radiology Quzhou People's Hospital Quzhou China; ^4^ Shenzhen Clinical Medical School of Southern Medical University Shenzhen China; ^5^ Department of Oncology Shenzhen Hospital of Southern Medical University Shenzhen China

**Keywords:** breast cancer survivors, CALM, chemotherapy, CRCI, rs‐fMRI

## Abstract

**Background:**

Chemotherapy related cognitive impairment (CRCI) is a type of memory and cognitive impairment induced by chemotherapy and has become a growing clinical problem. Breast cancer survivors (BCs) refer to patients from the moment of breast cancer diagnosis to the end of their lives. Managing Cancer and Living Meaningfully (CALM) is a convenient and easy‐to‐apply psychological intervention that has been proven to improve quality of life and alleviate CRCI in BCs. However, the underlying neurobiological mechanisms remain unclear. Resting‐state functional magnetic resonance imaging (rs‐fMRI) has become an effective method for understanding the neurobiological mechanisms of brain networks in CRCI. The fractional amplitude of low‐frequency fluctuations (fALFF) and ALFF have often been used in analyzing the power and intensity of spontaneous regional resting state neural activity.

**Methods:**

The recruited BCs were randomly divided into the CALM group and the care as usual (CAU) group. All BCs were evaluated by the Functional Assessment of Cancer Therapy Cognitive Function (FACT‐Cog) before and after CALM or CAU. The rs‐fMRI imaging was acquired before and after CALM intervention in CALM group BCs. The BCs were defined as before CALM intervention (BCI) group and after CALM intervention (ACI) group.

**Results:**

There were 32 BCs in CALM group and 35 BCs in CAU group completed the overall study. There were significant differences between the BCI group and the ACI group in the FACT‐Cog‐PCI scores. Compared with the BCI group, the ACI group showed lower fALFF signal in the left medial frontal gyrus and right sub‐gyral and higher fALFF in the left occipital_sup and middle occipital gyrus. There was a significant positive correlation between hippocampal ALFF value and FACT‐Cog‐PCI scores.

**Conclusions:**

CALM intervention may have an effective function in alleviating CRCI of BCs. The altered local synchronization and regional brain activity may be correlated with the improved cognitive function of BCs who received the CALM intervention. The ALFF value of hippocampus seems to be an important factor in reflect cognitive function in BCs with CRCI and the neural network mechanism of CALM intervention deserves further exploration to promote its application.

## 1 INTRODUCTION

Breast cancer is the most common type of cancer in women with malignancies.^1^ Treatments for breast cancer mainly include surgery, chemotherapy, HER2 targeted therapy and endocrine therapy based on hormonal status.^2^, ^3^ Among these methods, chemotherapy is an effective treatment for breast cancer.^4^, ^5^ Although systemic chemotherapy can prevent recurrence in breast cancer, its side effects seriously endanger the quality of life (QOL) of patients.^6^ In the clinic, nausea and myelosuppression are common side effects of chemotherapy.^7^ However, chemotherapy‐related cognitive impairment (CRCI) is often overlooked, but it is a major side effect that harms the QOL of patients.^8^ CRCI manifests as certain kinds of cognitive deficiencies, such as decrements in working memory.^9^ Although systemic therapy prolongs the survival of breast cancer patients, CRCI leads to many difficulties in life. CRCI also leads to a higher risk of stress from working and family.

Developing treatments to detect and alleviate CRCI is essential to enhancing breast cancer survivors' QOL.^10^ A recent study found that exercise can alleviate the severity of CRCI in breast cancer patients.^11^ However, the effect is not obvious, and the patient's health level has high requirements. Managing Cancer and Living Meaningfully (CALM) is a novel and brief psychological intervention^12^, ^13^ that has been proven to alleviate cognitive decline and improve QOL in breast cancer patients while reducing psychological distress.^14^ It provides a therapeutic purpose includes the following directions: communication with health care providers and symptom managements, relationships with close others and self‐changes, spiritual well‐being and the sense of meaning, mortality and future‐oriented concerns.^12^ Therefore, the role of CALM in alleviating CRCI has received extensive attention. However, the specific mechanism by which it exerts this effect is unclear, which limits the widespread acceptance and application of CALM interventions.

The identification of mechanisms that mediate the effects of psychological interventions are often explored through functional brain activity. Resting state functional magnetic resonance imaging (rs‐fMRI) is a technology for exploring human brain function.^15^ Functional interactions of brain regions can be explored by using rs‐fMRI.^16^ It has emerged as an important instrument for exploring the brain's response to external stimulus and various psychological interventions.^17^

Previous studies have been conducted to analyze the biological mechanism of CRCI by resting state fMRI.^18^, ^19^ Studies using RS‐fMRI have found the correlation between CRCI and changes in the left dorsolateral prefrontal cortex and central anterior gyrus function in breast cancer patients, and DTI imaging has been used to clarify the white matter changes caused by CRCI. A longitudinal resting state fMRI study completed by Zhang et al. found that the functional connection pattern of the left insula, temporal lobe and left inferior frontal gyrus to the hippocampus in breast cancer patients after chemotherapy was significantly changed.^20^ Another study found through brain network analysis that breast cancer patients after chemotherapy showed significantly increased global efficiency and abnormal central executive network (CEN) node characteristics and connections.^21^ Wang et al. used structural magnetic resonance to analyze the correlation between the reduction of cerebral cortex volume and thickness and CRCI in patients with non‐small cell lung cancer.^22^ Usually, amplitude of low‐frequency fluctuations (ALFF), fALFF (fractional ALFF) and degree centrality (DC) serves as common indicators in rs‐fMRI studies.^23^ Downregulated ALFF in specific regions may suggest dysregulated regional spontaneous neural function correlated with poorer cognitive performance.^24^ DC reflects the communication ability of a node in the functional network.^25^ The fALFF reduced the effects of physiological noise on basis of ALFF.^26^ These resting state fMRI studies have helped us understand the pathogenesis of CRCI from a functional imaging perspective of the brain.

The underlying mechanisms of the effect of the CALM intervention have yet to be fully elucidated. Therefore, our group focuses on fMRI‐based mechanisms for the CALM intervention. We designed this research to explore the changed ALFF brain regions between the BCI (before CALM intervention) group and the ACI (after CALM intervention) group with the purpose of identifying the potential brain regions that are correlated with CRCI and evaluating their ability to predict the intervention effect.

## 2 METHODS

### 2.1 Participants and baseline characteristics

Breast cancer survivors (BCs) were recruited in this research at the Second Hospital of Anhui Medical University. The inclusion criteria were as follows: (1) aged more than 20 to under 73 years; (2) breast cancer diagnosed in adulthood; (3) FACT‐Cog‐PCI score < 63^27^, ^28^; (4) The patient complains of cognitive impairment after receiving conventional‐dose taxane‐ or anthracycline‐based chemotherapy. Exclusion criteria included history of other cancers, psychological disorders and dementia. Finally, 32 BCs in CALM group and 35 BCs in CAU group completed the study. The demographic characteristics of patients who completed the overall study are presented in Table 1. This study was approved by the ethics committee of Anhui Medical University, and all included patients provided written informed consent.

**TABLE 1:** Comparison of demographic characteristics and clinical data of patients with breast cancer between the CALM group and the CAU group.

	CALM group	CAU group	*t*/*z*/*χ*	*p*
Age (years)	53.469 ± 7.462	51.229 ± 6.193	−1.341	0.184
Tumor stage				
I	4	7	1.338	0.732
II	14	17		
III	9	7		
IV	5	4		
Molecular classification				
Luminal A	4	6	1.488	0.713
Luminal B	14	18		
HER‐2 overexpression	10	9		
TNBC	4	2		
KPS				
80	13	11	0.615	0.433
90	19	24		

*Note*: Data are presented as the mean ± SD.

Abbreviations: CALM, Managing Cancer and Living Meaningfully; CAU, care as usual; KPS, Karnofsky Performance Status.

### 2.2 Intervention procedure

The CALM intervention is an original, systematic, tailored psychological intervention. It was designed by a team from the Princess Margaret Cancer Center, Toronto, Canada.^12^ In this research, BCs in CALM group participated in a 12‐week study with biweekly sessions for all six cycles of CALM interventions. The complete process takes place in a comfortable and quiet environment with soothing background music, and each session lasts approximately 30–60 min. It covers the following four domains: (1) symptom management and communication with health care providers; (2) changes in self and relations with close others; (3) spirituality, sense of meaning and purpose; (4) preparing for the future, sustaining hope and facing mortality (Supplementary Material).

### 2.3 Neuropsychological tests

The Functional Assessment of Cancer Therapy Cognitive Function (FACT‐Cog) is a self‐rating scale for assessing cancer patients' cognitive function.^29^ The version number of FACT‐Cog used in this research is Version: 3. This assessment is administered by interviewing patients. This scale can be used to assess cancer patients' cognitive function with 37 items, including four main dimensions: perceived cognitive impairment (FACT‐Cog‐PCI), comments from others (FACT‐Cog‐Oth), perceived cognitive ability (FACT‐Cog‐PCA), and cognitive changes on QOL (FACT‐Cog‐QOL). Each item is scored on a scale of 0–4, with a total of five response options. The status of cognitive function can be reflected by the score. The English version of the scale has been shown to have good validity and reliability.^30^ Studies have shown that the Chinese version of the FACT‐Cog also has good validity and reliability for Chinese breast cancer survivors, and it can be used to clinically evaluate the cognitive function status of Chinese breast cancer survivors.^31^, ^32^

### 2.4 MRI data acquisition

The MRI data were scanned by a Siemens 3.0 T scanner (Germany) with a 16‐channel head coil at the Second Affiliated Hospital of Anhui Medical University. The whole scan took approximately 30 min. The rs‐fMRI image was scanned by a gradient‐recalled echo‐planar imaging (GRE‐EPI) pulse sequence. The rs‐fMRI data were obtained as follows: TE = 25 ms, TR = 2000 ms, acquisition matrix = 64 × 64, FA = 90°, FOV = 240 × 240 mm, thickness = 4.0 mm, gap = 0 mm, NEX = 1.0, slice number = 36, and time points = 240. The T1‐weighted 3D 3D‐SPGR images were obtained with the following parameters: TR = 1900 ms, TE = 2.48 ms, FA = 9°, acquisition matrix = 256 × 256, FOV = 240 × 240 mm, thickness = 1.0 mm, gap = 0 mm, slice number = 176, and NEX = 1.0. During the MRI scans, all patients were required to keep their eyes closed and avoid distraction during the scanning.

### 2.5 Data preprocessing

In this study, we used DEPASF 4.3 Edition (http://rfmri.org/dpabi) based on MATLAB 2013b for preprocessing and statistical analysis of fMRI data. The first step in the operation is to convert the Digital Imaging and Communication in Medicine (DICOM) data images into Neuroimaging Information Technology Initiative (NIFTI) images. The first 10 volumes were discarded as a sign of subject adaptation to the scan. The next step was slice time and motion correction head, and the MRI images were slice time corrected and realigned with the first volume. Then, in the step of spatial normalization, we spatially normalize the reconstructed MRI images according to the T1 structural images of each subject. All images were subsequently resampled to a 3‐mm isotropic voxel. We then perform smoothing, process the image with a 4‐mm full‐width half‐maximum Gaussian kernel, and spatially smooth the resampled image to reduce spatial noise.^33^, ^34^, ^35^ All subjects' head motion during scans will be examined to determine if translation exceeds 2 mm or rotation exceeds 2°; in such cases, the data will be discarded for quality control.

### 2.6 DC and GMV analyses

DC is an analysis method based on the voxel level, which reflects the functional importance of a voxel in the brain network by analyzing the number of instant functional connections between a voxel and other voxels in the brain, and there are many studies showing that DC has higher reliability and repeatability than other indicators. SPM software (version 12.0) was used to perform linear and nonlinear transformations of high‐resolution T1 structural images to complete spatial normalization and segment the tissues into white matter, gray matter and cerebrospinal fluid, while obtaining the processed GMV, which was smoothed using a Gaussian image core with 6 mm full width and half a maximum.

### 2.7 ALFF and fALFF analyses

ALFF is a new neurophysiological marker of fMRI to assess brain function. This marker was proposed by Zang et al. It can be used to detect the local intensity of spontaneous fluctuations in BOLD signals to pinpoint the spontaneous neural activity and physiological state of the brain in a specific region.^36^ Electrophysiological studies^37^ have found that ALFF may reflect the features of the brain. Previous studies also proposed that internal resting state activity reflects specific brain circuits that participate in cognitive work.^38^ The analysis of the ALFF indicator is as follows. To calculate ALFF, in the power spectrum range of 0.01–0.08 Hz, each voxel of the time series is averaged by the fast Fourier transform of its square root. ALFF values were subtracted from the mean and divided by the whole‐brain voxel bias, converted to a z‐distribution, and finally normalized. The obtained results were then processed with a 0.01–0.08 Hz bandpass filter. The fALFF is a normalized version of ALFF, and has often been used in analyzing the power and intensity of spontaneous regional resting state neural activity.

### 2.8 Statistical analysis

SPSS 21.0 was used to analyze demographic data. Data are presented as the mean ± standard deviation (SD). The chi‐square test and Student's *t*‐test were used to compare differences in demographic data. The paired *t*‐test and the paired Wilcoxon signed‐rank test were performed for normally and nonnormally distributed continuous variable, respectively. The independent samples *t*‐test and Mann–Whitney *U*‐test were performed to compare normally and nonnormally distributed continuous variables, respectively. DPARSF software was used for statistical analysis, and two‐sample *T* test was used for inter‐group analysis. After correction (*p* < 0.05), fALFF and DC difference maps were obtained after comparison between the two groups. The permutation test was applied to calculate significance, and threshold‐free cluster enhancement (TFCE) joint correction was subjected to multiple correction analysis. All results were corrected for multiple comparisons using TFCE FWE at *p* < 0.05.

## 3 RESULTS

### 3.1 Patients

Baseline characteristics of patients who completed the overall study are presented in Table 1.

### 3.2 CRCI assessment

**FIGURE 1:**
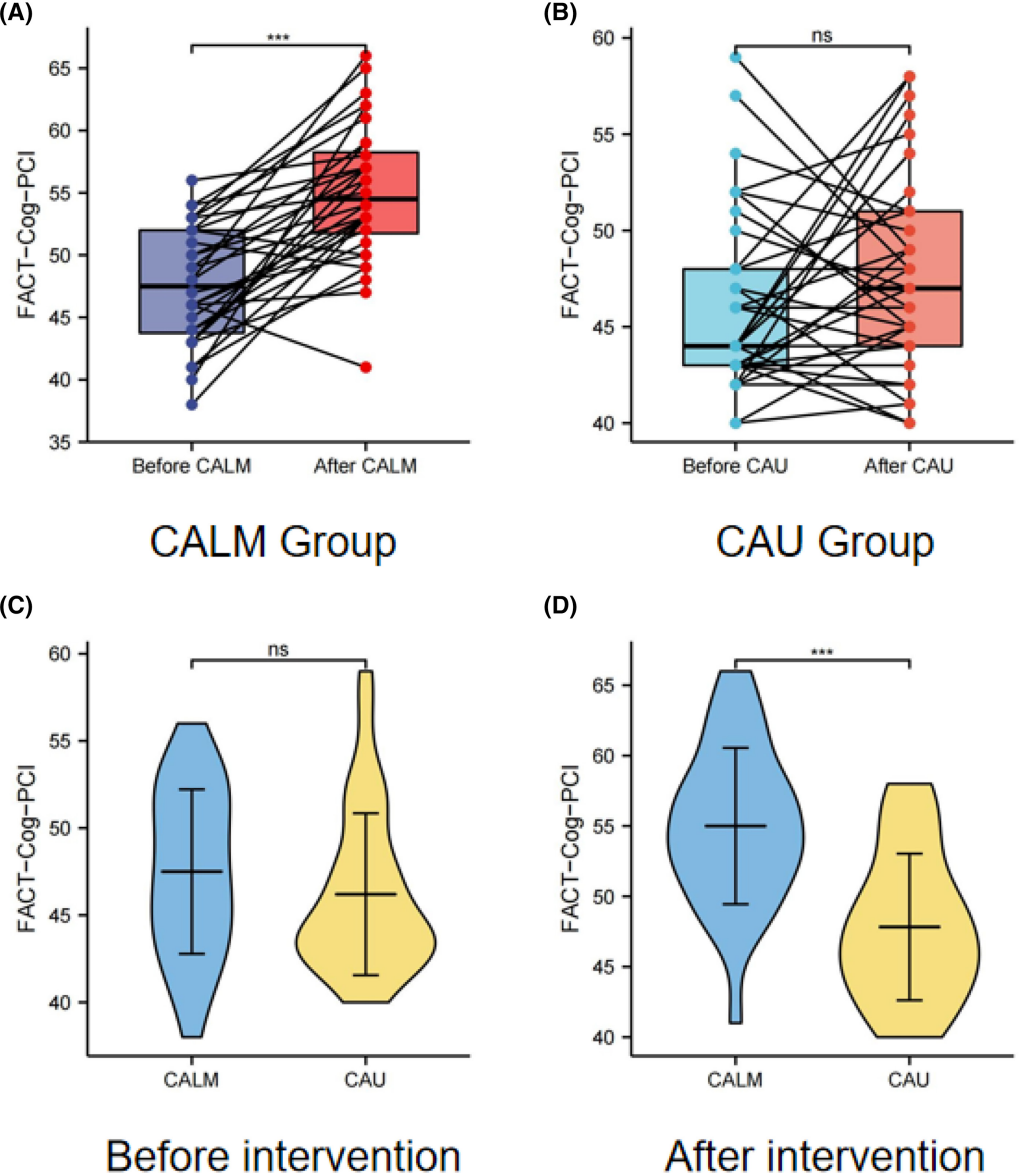
Changes in cognitive function of breast cancer survivors before and after the CALM/CAU intervention.

The FACT‐Cog‐PCI score of CALM patients after CALM intervention was 55.0 ± 5.53, which was significantly better than 47.8 ± 5.21 of CAU patients (Figure 1 and Table 2). There was no significant difference in FACT‐Cog‐PCI score before and after CAU (Figure 1B). Such results indicate the significant effect of the CALM intervention in improving perceived cognitive function of patients with breast cancer.

**TABLE 2:** Changes in cognitive function of breast cancer survivors before and after the CALM/CAU intervention.

	CALM group	CAU group	*t*/*z*	*p*
FACT‐Cog‐PCI				
Before CALM/CAU	47.5 ± 4.72	46.2 ± 4.65	−1.387^a^	0.166
After CALM/CAU	55.0 ± 5.53	47.8 ± 5.21	−7.171	<0.001

Abbreviations: CALM, Managing Cancer and Living Meaningfully; CAU, care as usual.

^a^ Analyzed using the Mann–Whitney *U*‐test.

### 3.3 Changes in gray matter volume (GMV) before and after CALM intervention

**FIGURE 2:**
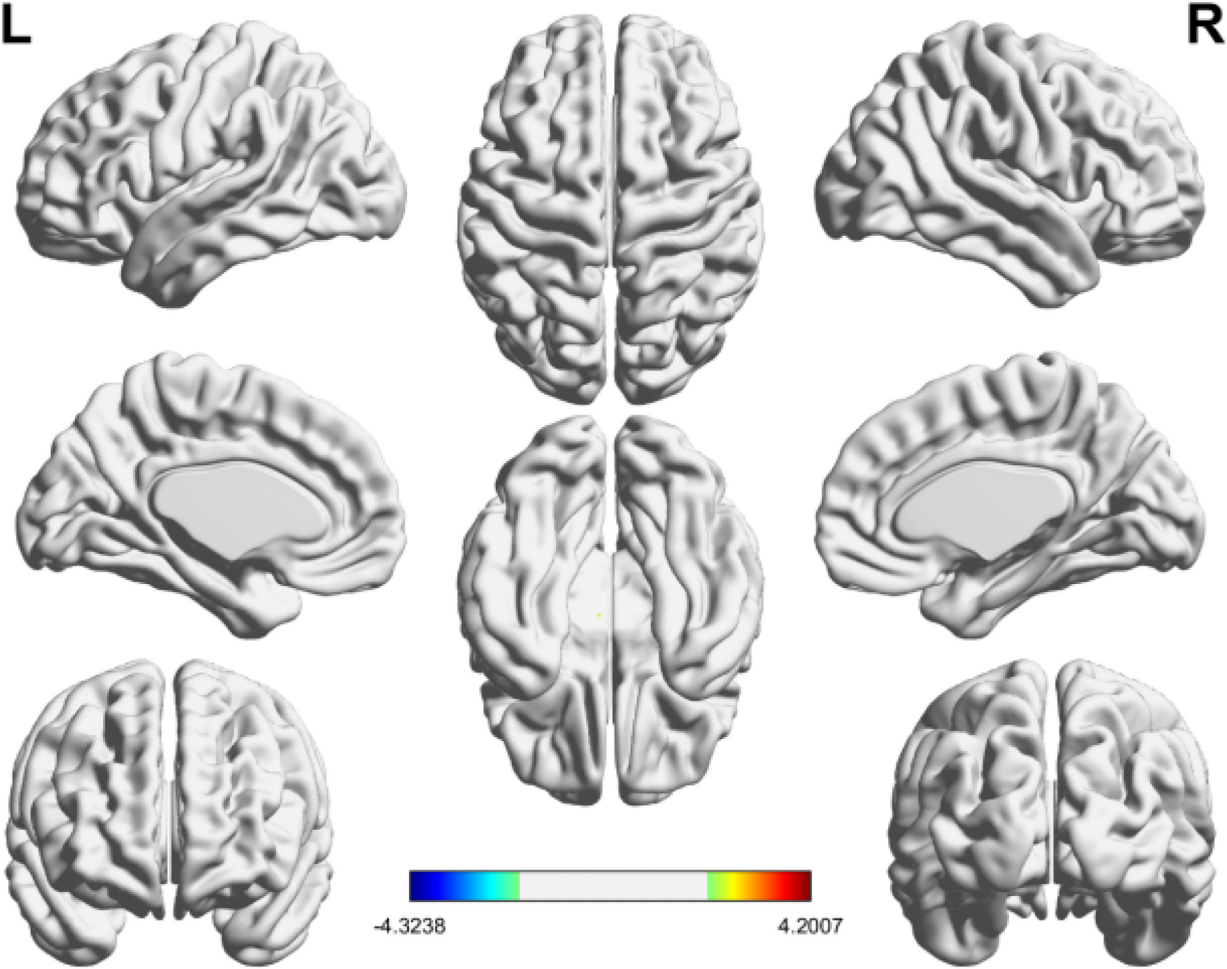
Changes in gray matter volume before and after the CALM intervention.

For the BCs in CALM group, there were no significant changes in gray matter volume before and after CALM intervention (Figure 2).

### 3.4 Changes of fALFF before and after CALM intervention

**FIGURE 3:**
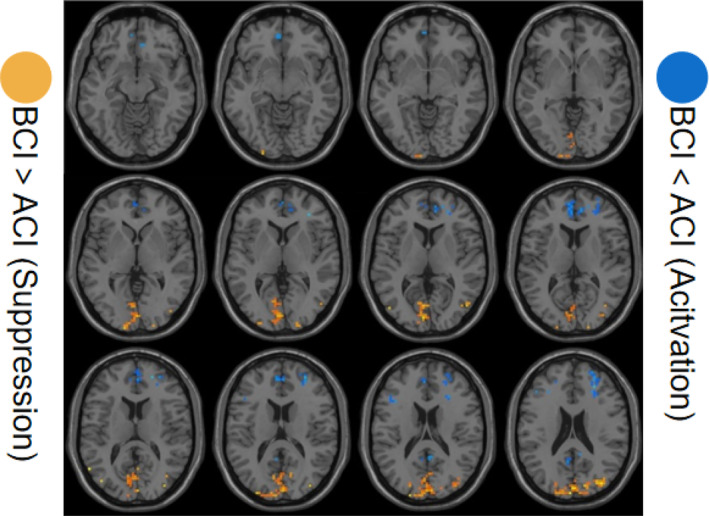
Comparison of fALFF values between the BCI and ACI groups. BCI, before CALM intervention; ACI, after CALM intervention.

The fALFF differences were analyzed to assess activated or suppressed brain regions after CALM intervention. The results showed activated resting‐state fALFF in left occipital lobe, occipital_sup and right middle occipital gyrus. And suppressed resting‐state fALFF in left medial frontal gyrus, right sub‐gyral and medial frontal gyrus (Table 3 and Figure 3).

**TABLE 3:** Regions showing significant differences in fALFF values before and after CALM.

Brain regions	L/R	MNI coordinate			Voxels	*T* value
		X	Y	Z		
BCI<ACI (activation)						
Cluster 3‐occipital lobe	L	−24	−99	−9	2	3.9695
Cluster 4‐occipital_sup	L	0	−87	21	53	5.3231
Cluster 5‐calcarine	R	18	−96	3	3	3.53
Cluster 6‐middle occipital gyrus	R	42	−81	0	1	3.6349
Cluster 8‐middle occipital gyrus	L	−27	−96	3	2	3.4513
Cluster 9‐middle occipital gyrus	R	48	−72	6	4	3.781
BCI>ACI (suppression)						
Cluster 1‐medial frontal gyrus	R	6	36	−15	4	−3.9352
Cluster 2‐frontal_med_orb	L	−6	45	−8	7	−3.8096
Cluster 7‐medial frontal gyrus	L	−3	45	18	30	−3.7933
Cluster 10‐sub‐gyral	R	33	39	3	1	−4.7028

Abbreviations: ACI, after CALM intervention; BA, Bordmann area; BCI, before CALM intervention; MNI coordinates, Montreal Neurological Institute coordinates; L, left; R, Right.

### 3.5 Changes of DC before and after CALM intervention

**FIGURE 4:**
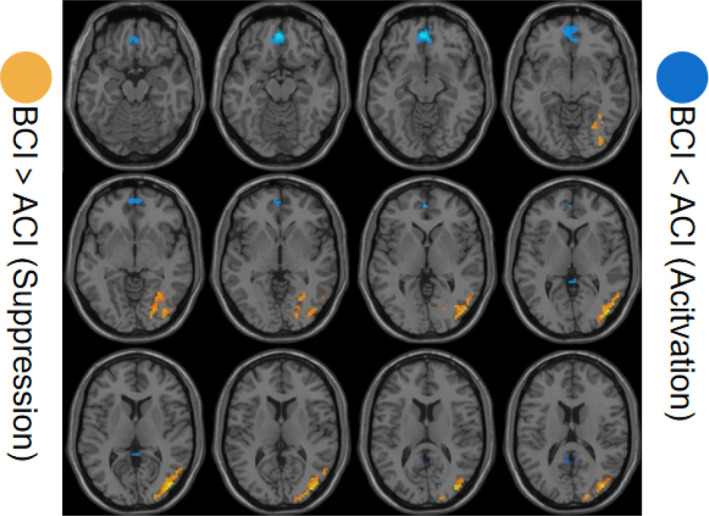
Comparison of DC values between the BCI and ACI groups. BCI, before CALM intervention; ACI, after CALM intervention.

The DC differences were analyzed to assess activated or suppressed brain regions after CALM intervention. The results showed activated resting‐state DC in right lingual gyrus, temporal_inf and temporal lobe. And suppressed resting‐state DC in left rectal gyrus, precuneus and right cingulum_post (Table 4 and Figure 4).

**TABLE 4:** Regions showing significant differences in DC values before and after CALM.

Brain regions	L/R	MNI coordinate			Voxels	*T* value
		X	Y	Z		
BCI<ACI (activation)						
Cluster 3‐cerebelum_6	R	27	−54	−21	2	4.8599
Cluster 4‐lingual gyrus	R	24	−78	−6	16	4.4487
Cluster 5‐temporal_inf	R	39	−60	−9	1	3.6246
Cluster 6‐temporal lobe	R	36	−51	−9	2	4.3757
Cluster 7‐occipital_mid	R	42	−81	3	134	6.6177
BCI>ACI (suppression)						
Cluster 1‐rectal gyrus	L	−3	36	−21	3	−4.1758
Cluster 2‐frontal_med_orb	L	−6	48	−12	44	−6.6072
Cluster 8‐cingulum_post	R	3	−42	3	2	−4.2282
Cluster 9‐precuneus	L	−6	−42	33	110	−5.8558
Cluster 10‐frontal_sup	R	24	42	36	72	−5.0245

Abbreviations: ACI, after CALM intervention; BCI, before CALM intervention; L, left; MNI coordinates, Montreal Neurological Institute coordinates; R, Right.

### 3.6 The correlation between ALFF value of hippocampus and FACT‐Cog‐PCI

**FIGURE 5:**
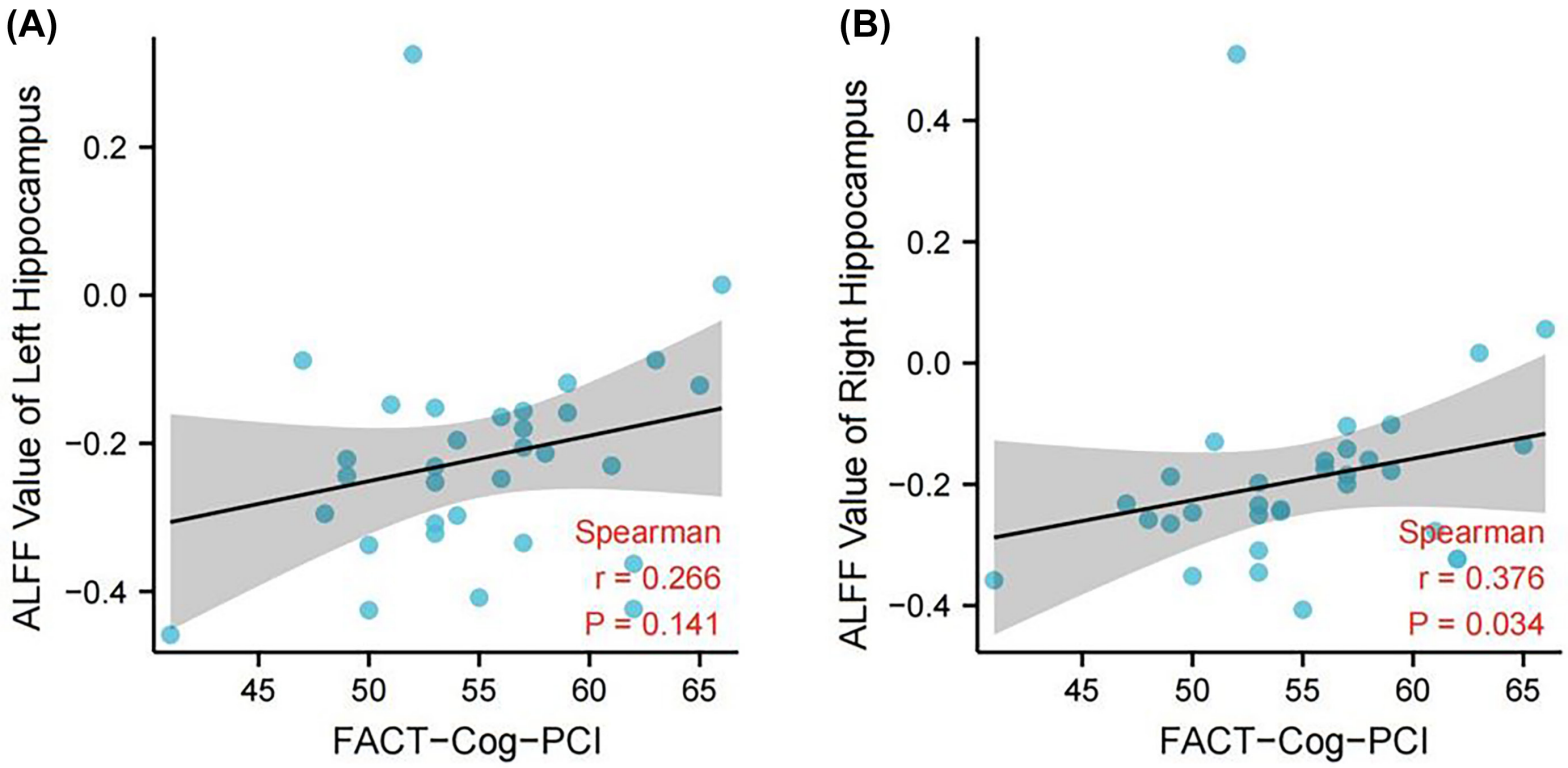
Correlation analysis between FACT‐Cog‐PCI scores and hippocampal ALFF values. (A). The correlation between FACT‐Cog‐PCI scores and left hippocampal ALFF values; (B). The correlation between FACT‐Cog‐PCI scores and right hippocampal ALFF values.

As shown in Figure 5, the correlation between the FACT‐Cog‐PCI score and right hippocampus ALFF value was significant (*R* = 0.376, *p* = 0.034). The correlation between left hippocampus ALFF value and the FACT‐Cog‐PCI score was not statistically significant (*R* = 0.266, *p* = 0.141).

**FIGURE 6:**
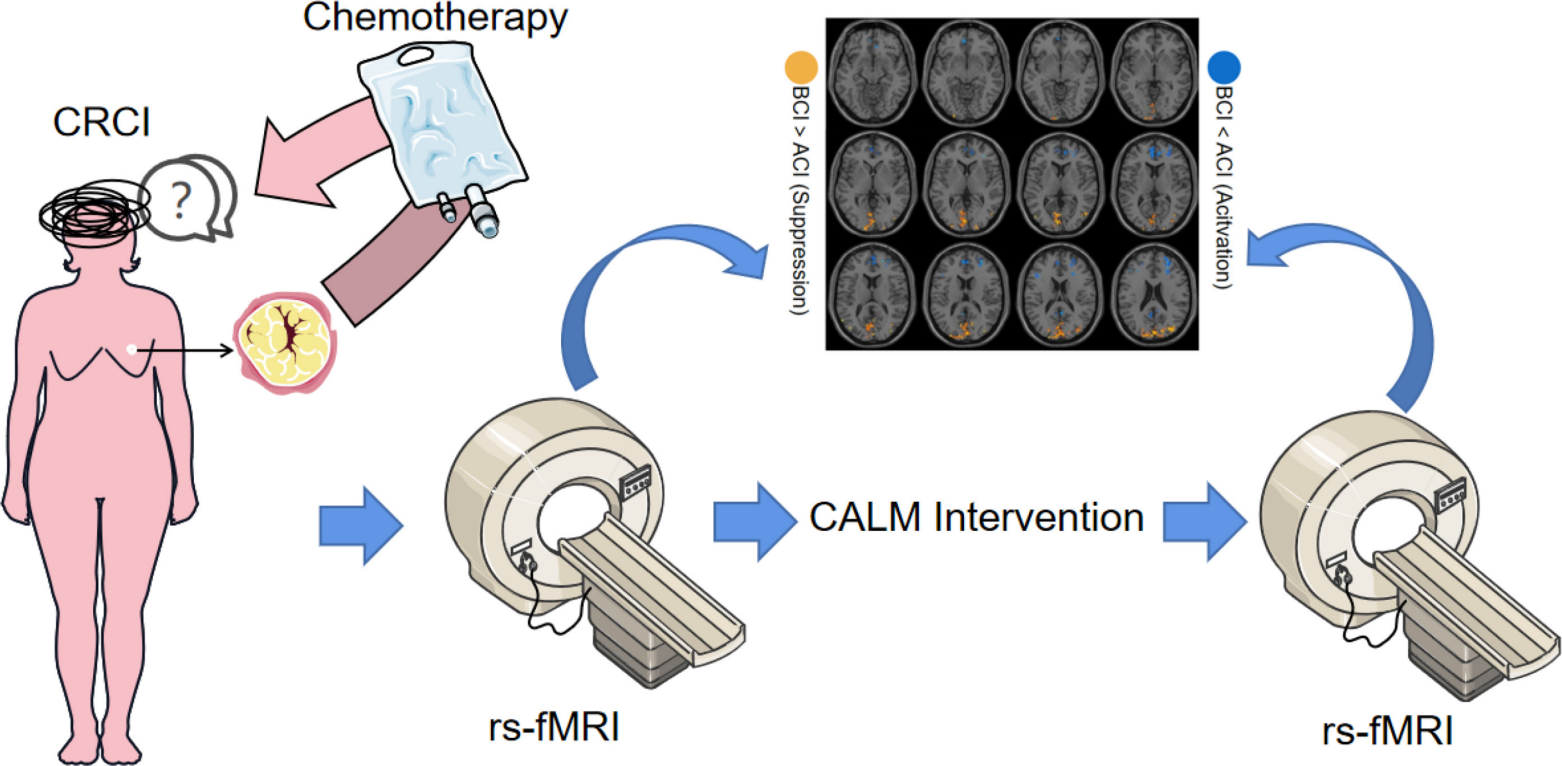
Overview schematic of the study. BCI, before CALM intervention; ACI, after CALM intervention; CRCI, chemotherapy related cognitive impairment; rs‐fMRI, resting‐state functional magnetic resonance imaging.

## 4 DISCUSSION

In this study, we found that the CALM intervention alleviated cognitive dysfunction in BCs after chemotherapy. Additionally, the rs‐fMRI functional indicators of BCs before and after the intervention also showed significant differences. These results indicate that CALM intervention may ameliorate cognitive decline in BCs by modulating the activation or inhibition of signals in brain regions (Figure 6).

After CALM intervention, the patient's cognitive function was significantly improved. In breast cancer patients, the side effects of chemotherapeutics and cancer itself on cognitive function have been implied to impact many brain areas involved in processing speed, attention, executive functions, and memory.^39^ Thus, implementing the CALM intervention to improve cognitive function may also affect these brain regions. Therefore, we used resting‐state fMRI to analyze specific indicators.

The scientific community currently believes that ALFF can reflect the regional activity of the brain.^40^, ^41^ By analyzing the ALFF indicator, Jing et al. found that exercise can significantly improve cognitive function and modulate gray matter volume in the cingulate cortex.^42^ Our findings are broadly consistent with their opinion. Previous research explored the relationship between temporal lobe connectivity and cognitive function in early Alzheimer's disease.^43^ They found that cognitively normal individuals were mainly characterized by changed functional connectivity in the temporal lobe. Thus, functional connectivity of the temporal lobe could serve as a stage‐specific functional marker in early Alzheimer's disease. In this study, breast cancer patient changes in cognitive function may also be associated with activation of fALFF indictors in the temporal lobe. A large sample study recently found that the subgyral lobe of the temporal lobe functions as a very important brain region in human working memory.^44^ This result is consistent with our fALFF analysis. In our results, we also observed inhibition of ALFF signaling for the occipital lobe and middle occipital gyrus. In fact, studies have found that the reduction in occipital gray matter volume is closely related to cognitive dysfunction in Parkinson's disease.^45^ Our study also found that the fALFF signal of the occipital lobe changed after CALM intervention. Research on the middle occipital gyrus also found that this brain region has abnormal signals in the brain functional connections of female depressed patients.^46^

The hippocampus has been shown to play an important role in cognitive function.^47^, ^48^ The interaction between CRCI and hippocampal alterations has received extensive attention.^49^ This study found that the changes in cognitive function in breast cancer patients after the CALM intervention were correlated with the ALFF value of the hippocampus. Therefore, we once again confirmed the important role of the hippocampus in cognitive regulation. Future studies should explore the correlation of altered hippocampal function with different cognitive abilities, such as digit span, prospective memory, and retrospective memory.

Combining the results above, we found that the activation or inhibition of fALFF signaling in different brain regions is related to the physiological cognitive function of the subjects in this study. After CALM intervention, the fALFF signal intensity in different brain regions was changed. And these changes were correlated with changes in subjects' cognitive function, which suggesting that we should explore the effects of CALM intervention on these brain regions from a clinical perspective. By analyzing the correlation between FACT‐Cog‐PCI scores and hippocampal ALFF, we found that the signal intensity of ALFF in hippocampal gyrus is positively correlated with cognitive function. From the clinical physiological point of view to observe the changes in subjects, we found that the hippocampus is an important brain cognitive structure, and CALM may improve the cognitive function of patients by regulating its function. The potential link between changes of ALFF signal in Cingulate Gyrus, Temporal Lobe, Subgyral, Occipital Lobe, Middle Occipital Gyrus regions and changes in patients' cognitive function is an important direction to promoting CALM intervention.

Currently, CRCI can remain a severe symptom in up to 35% of breast cancer survivors and negatively affects their QOL.^50^ CALM was confirmed as a practical intervention that alleviate CRCI.^14^ Understanding neurobiological mechanisms of alleviate CRCI is important to define the characteristics of beneficial CALM intervention outcome,^51^ which also is the specific clinical application value of the results in this study. Future fMRI analysis could show the difference in signal strength in different brain regions during CALM intervention. And it would be very informative to predict the effectiveness of CALM intervention alleviating CRCI.^52^ The results in this study also provide new insights into the brain science mechanisms of CALM that alleviate CRCI, providing information on specific brain areas as well as evidence of their mechanism of action via CALM.^53^

There are also some shortcomings of this study. Given the important changes of the cognition with age, the age range of selected subjects should be limited as much as possible to avoid age‐related confounding factors.^54^ From the perspective of fMRI analysis, this was an observational study that examined ALFF outcomes before and after CALM intervention. Since this is a pilot study, only patients in the CALM group received fMRI acquisition analysis before and after the intervention. In future studies, fMRI data should be collected from both the CALM group and the usual care group for comparative analysis. Given the temporal variation of cognitive function, future research should compare both groups over time. Compared to FACT‐Cog‐PCI, MMSE is not measuring cognitive functioning deeply. Future studies should use a variety of neuropsychological tests to improve the study and obtain more in‐depth cognitive assessment results. The chemotherapeutic regime and cycle of enrolled patients should also be tightly controlled to obtain more convincing results in future. Some of the patients enrolled were treated with endocrine drugs, although a previous RCT study in JCO noted that BCs receiving chemotherapy had significantly heavier CRCI symptoms than those treated with endocrine therapy alone, the impact of hormonal therapy use on results still need to be considered in future studies.^29^, ^55^ The sample size of patients with breast cancer is limited. Future research needs to enroll more patients with breast cancer to enhance the validity and reliability of conclusion.

## 5 CONCLUSION

CALM intervention may have an effective function in alleviating CRCI of BCs. The altered local synchronization and regional brain activity may be correlated with the improved cognitive function of BCs who received the CALM intervention. The ALFF value of hippocampus seems to be an important factor in reflect cognitive function in BCs with CRCI and the neural network mechanism of CALM intervention deserves further exploration to promote its application.

## AUTHOR CONTRIBUTIONS

**Senbang Yao:** Conceptualization (lead); formal analysis (lead); software (equal); validation (equal); writing – original draft (lead). **Qinqin Zhu:** Formal analysis (lead); software (lead); visualization (lead). **Qianqian Zhang:** Conceptualization (lead); investigation (lead); supervision (lead). **Yinlian Cai:** Conceptualization (equal); methodology (equal); resources (equal). **Shaochun Liu:** Formal analysis (equal); methodology (equal); project administration (equal). **Lulian Pang:** Formal analysis (equal); investigation (equal); validation (equal). **Yanyan Jing:** Data curation (equal); methodology (equal); software (equal). **Xiangxiang Yin:** Data curation (equal); project administration (equal). **Huaidong Cheng:** Funding acquisition (lead); resources (lead).

## FUNDING INFORMATION

Funding was supplied by the National Natural Science Foundation of China (No. 81872504).

## CONFLICT OF INTEREST STATEMENT

The authors declare no conflicts of interest, and the publication of this manuscript was approved by all authors.

## ETHICS STATEMENT

All procedures performed involving human participants were in accordance with the ethical standards of the institutional and national research committee and with the 1964 Helsinki Declaration and its later amendments or comparable ethical standards. This study was approved by the ethics committee of Anhui Medical University (2018_81872504), and all included patients provided written informed consent.

## Supporting information


Supplementary Material

## Data Availability

The data that support the study may be available upon request from the corresponding author.
