# Supplementary figures and images for: Managing Cancer and Living Meaningfully (CALM) alleviates chemotherapy related cognitive impairment (CRCI) in breast cancer survivors: A pilot study based on resting‐state fMRI
Source: Cancer Med. 2023 Jul 6;12(15):16231–42. doi: 10.1002/cam4.6285 (PMC10469649; doi:10.1002/cam4.6285)

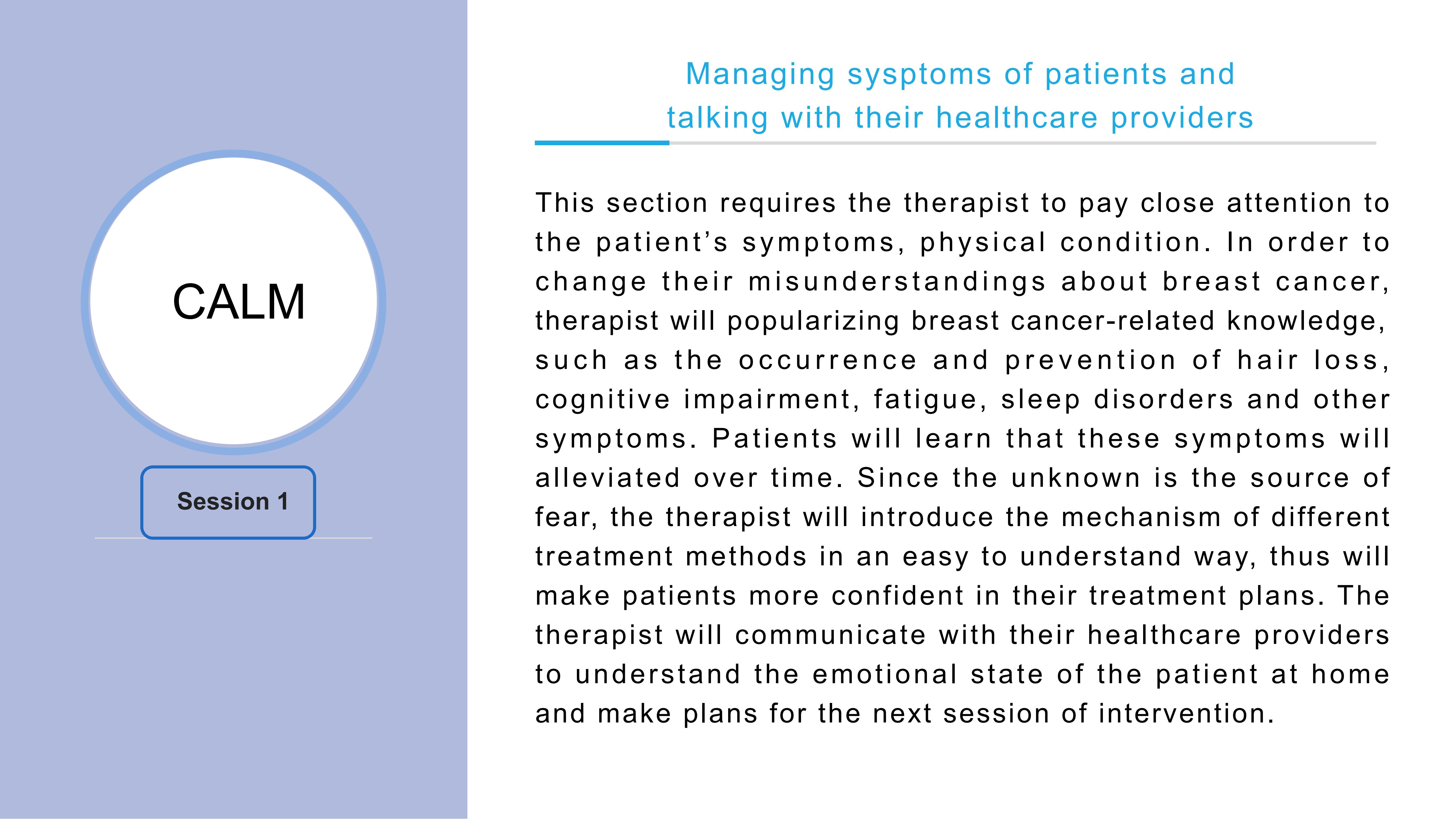

Supplement: Supplementary file 1 — Supplementary Material [file CAM4-12-16231-s001.zip › Supplementary Material_01.jpg]

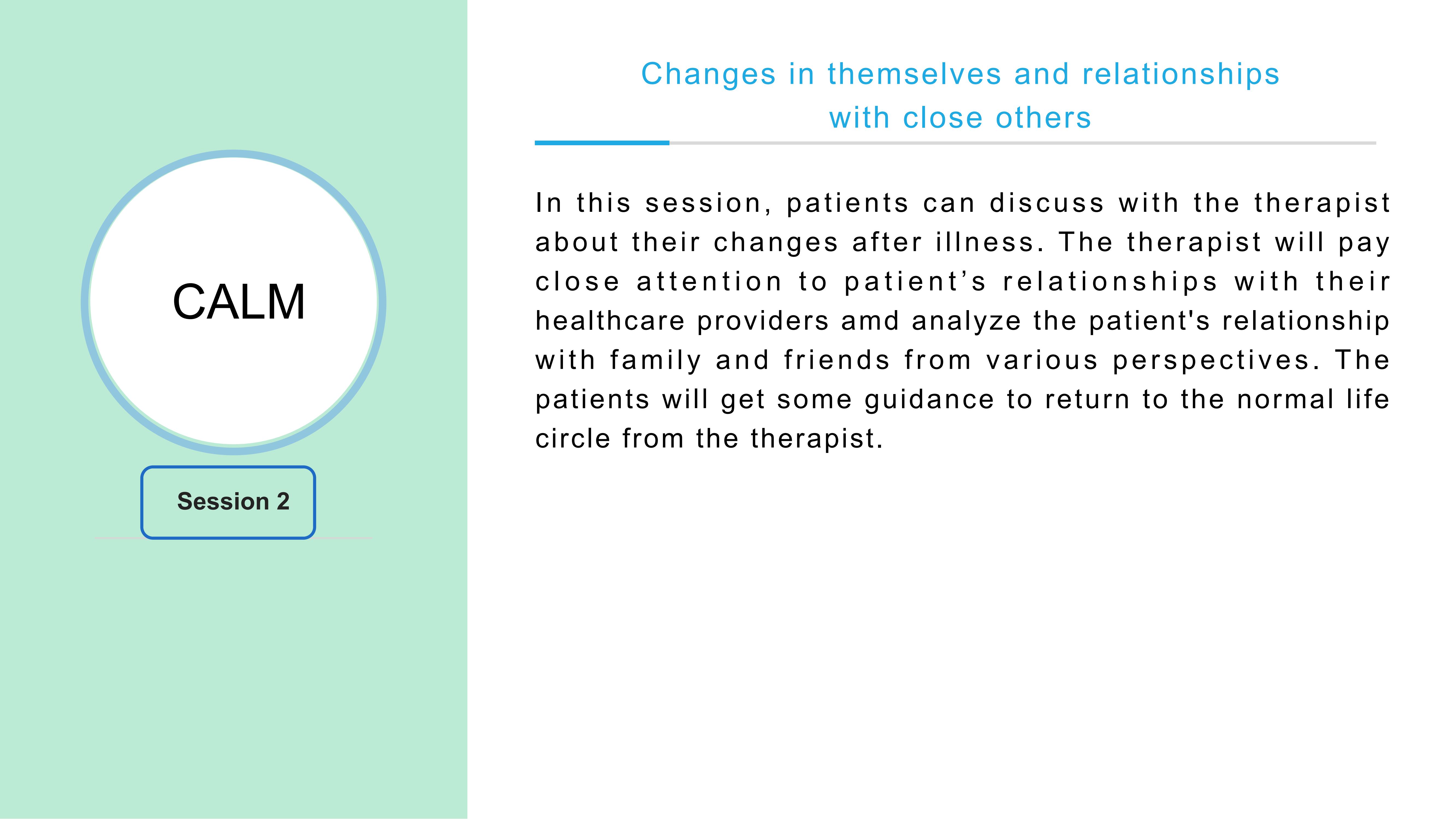

Supplement: Supplementary file 1 — Supplementary Material [file CAM4-12-16231-s001.zip › Supplementary Material_02.jpg]

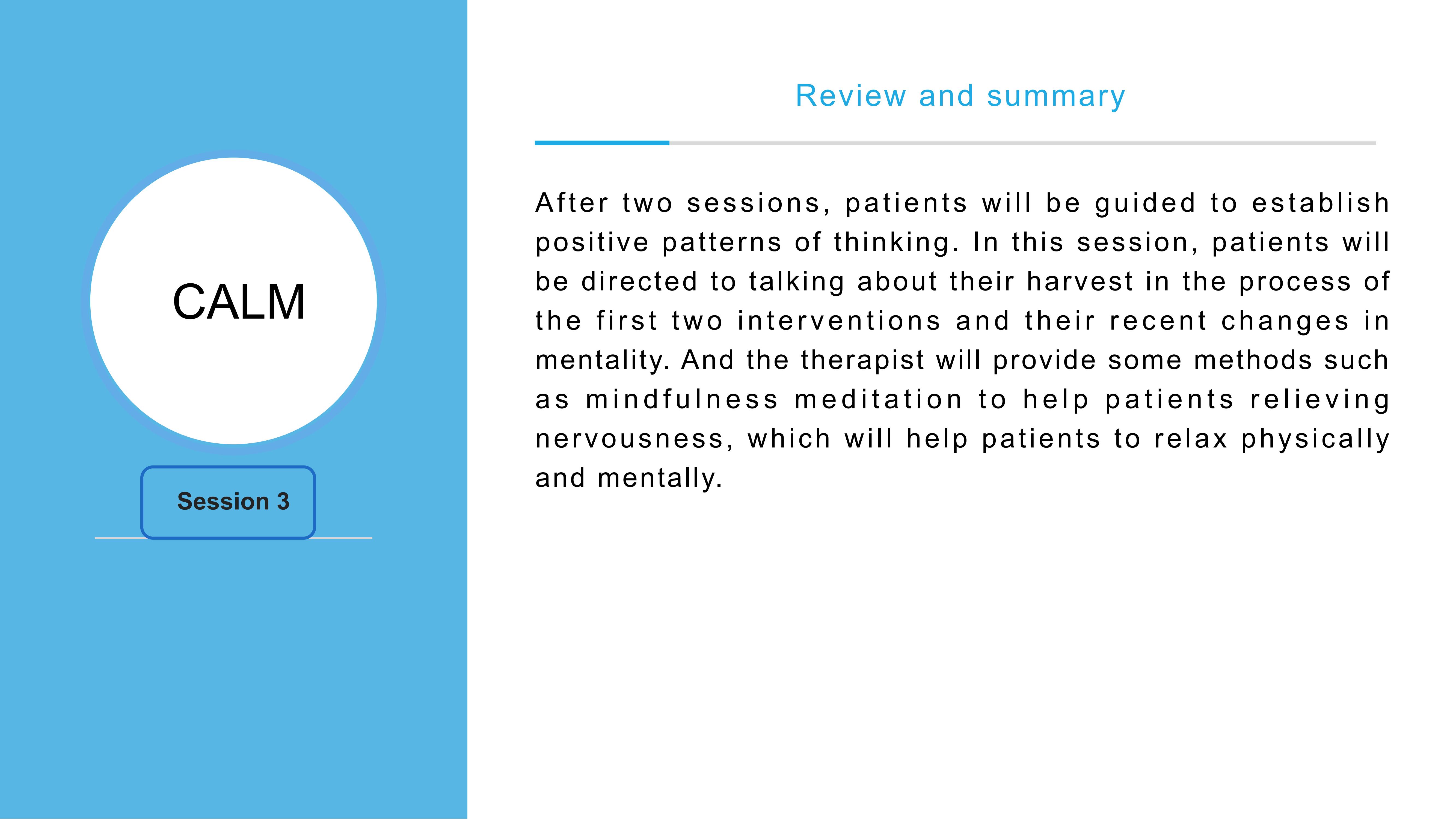

Supplement: Supplementary file 1 — Supplementary Material [file CAM4-12-16231-s001.zip › Supplementary Material_03.jpg]

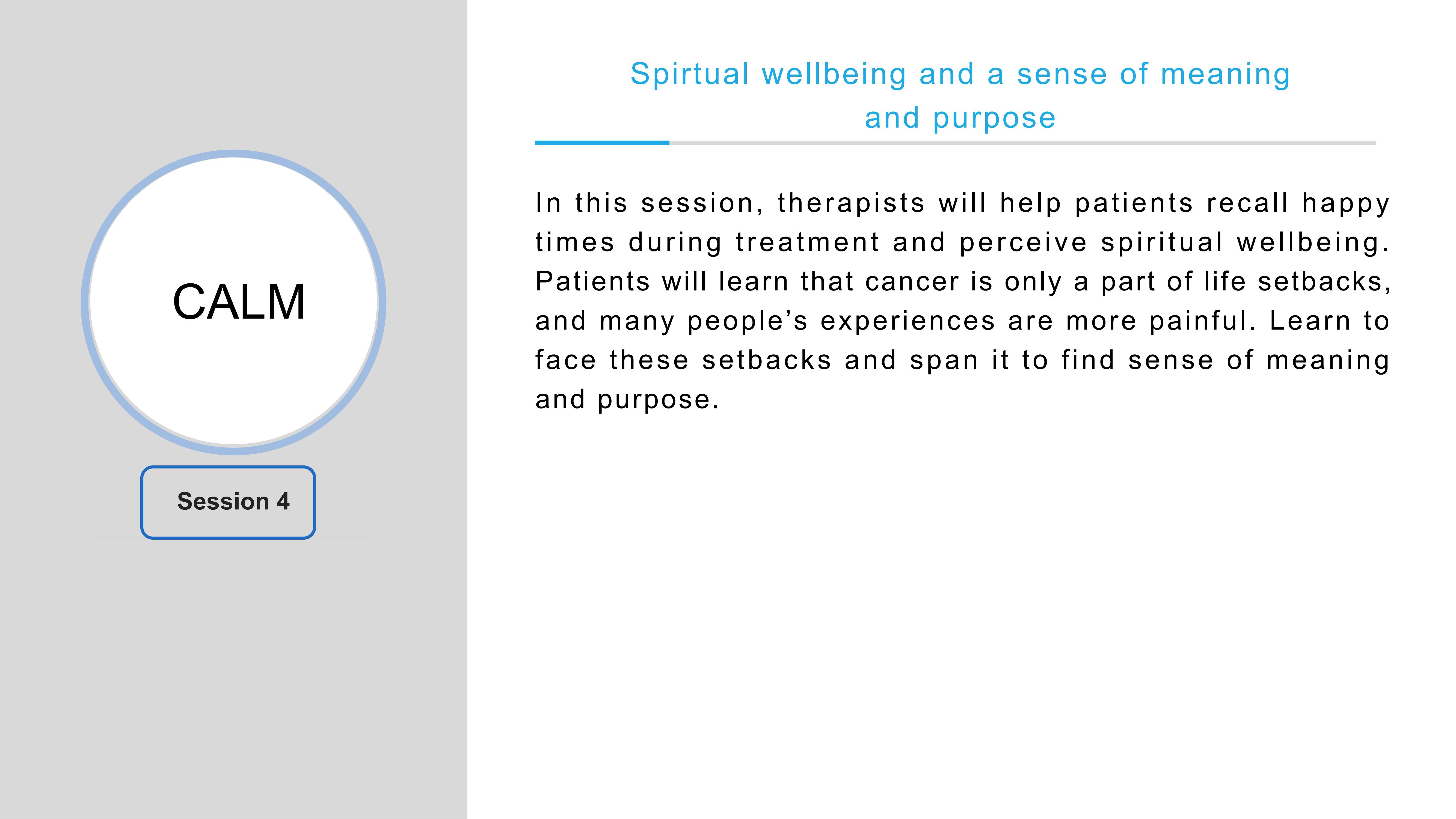

Supplement: Supplementary file 1 — Supplementary Material [file CAM4-12-16231-s001.zip › Supplementary Material_04.jpg]

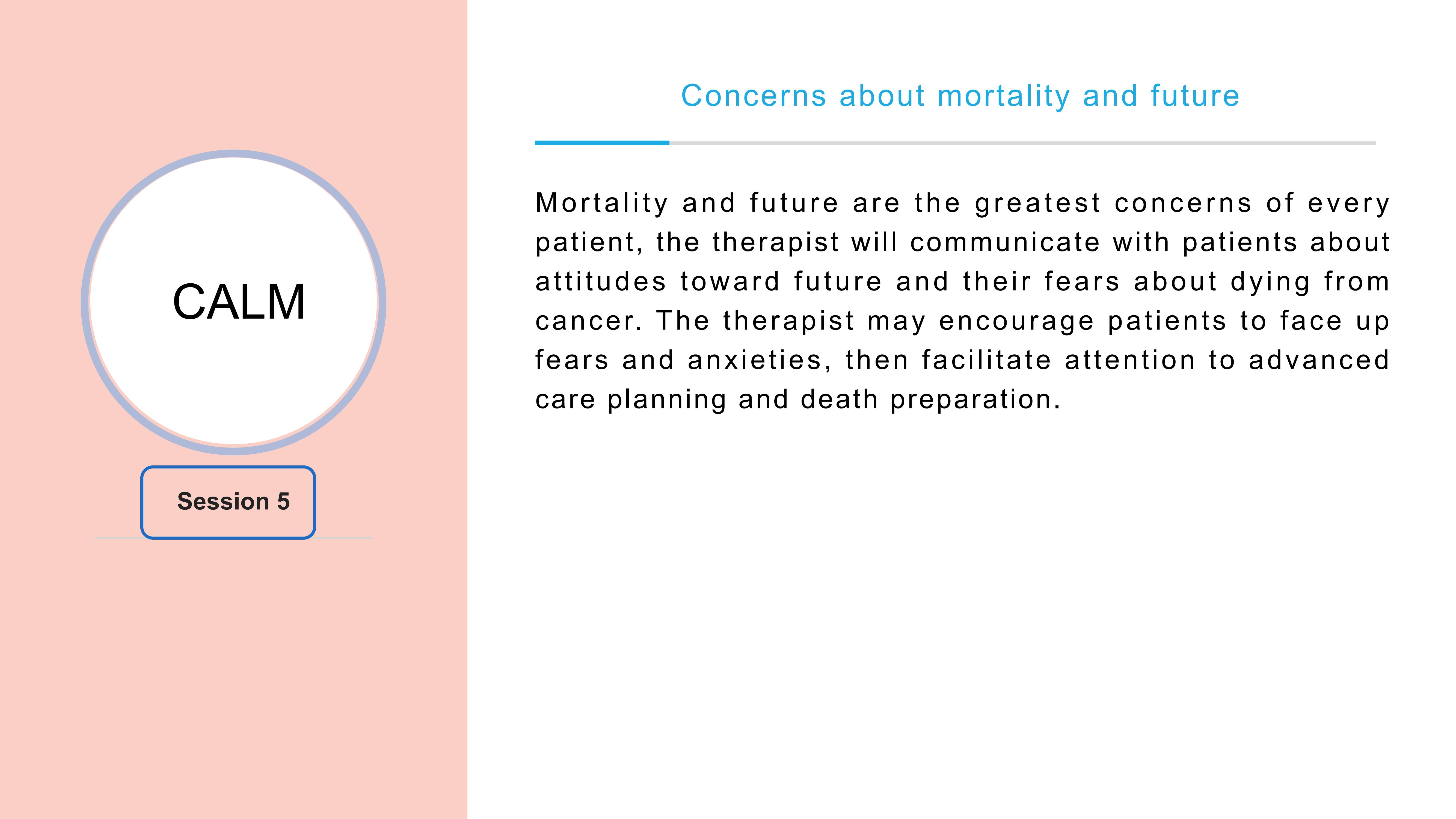

Supplement: Supplementary file 1 — Supplementary Material [file CAM4-12-16231-s001.zip › Supplementary Material_05.jpg]

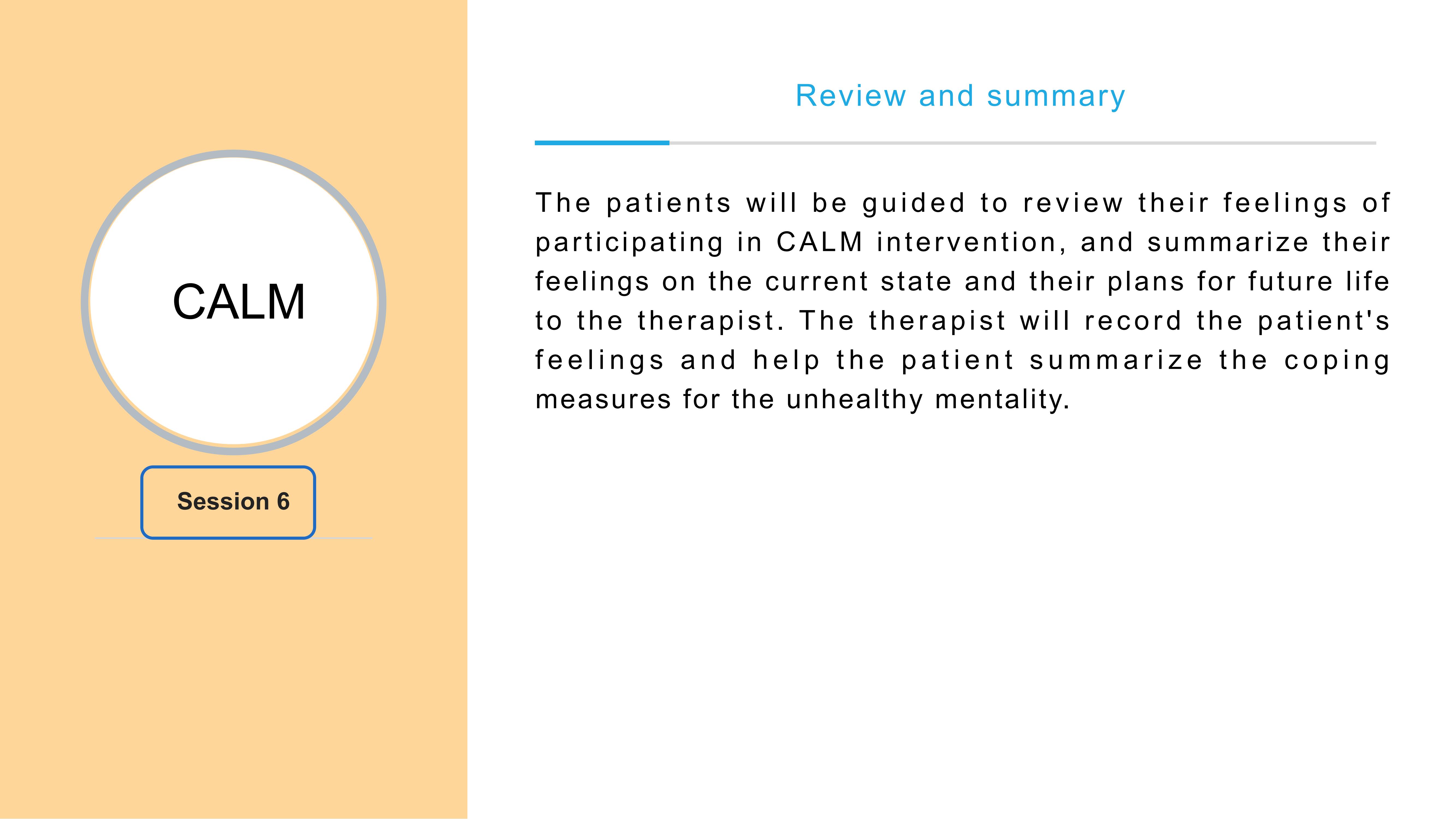

Supplement: Supplementary file 1 — Supplementary Material [file CAM4-12-16231-s001.zip › Supplementary Material_06.jpg]
